# e-Fungi: a data resource for comparative analysis of fungal genomes

**DOI:** 10.1186/1471-2164-8-426

**Published:** 2007-11-20

**Authors:** Cornelia Hedeler, Han Min Wong, Michael J Cornell, Intikhab Alam, Darren M Soanes, Magnus Rattray, Simon J Hubbard, Nicholas J Talbot, Stephen G Oliver, Norman W Paton

**Affiliations:** 1 School of Computer Science, The University of Manchester, Manchester, M13 9PL, UK; 2 Faculty of Life Sciences, The University of Manchester, Manchester, M13 9PT, UK; 3 School of Biosciences, University of Exeter, Exeter, EX4 4QD, UK; 4 Department of Biochemistry, University of Cambridge, Cambridge CB2 1GA, UK

## Abstract

**Background:**

The number of sequenced fungal genomes is ever increasing, with about 200 genomes already fully sequenced or in progress. Only a small percentage of those genomes have been comprehensively studied, for example using techniques from functional genomics. Comparative analysis has proven to be a useful strategy for enhancing our understanding of evolutionary biology and of the less well understood genomes. However, the data required for these analyses tends to be distributed in various heterogeneous data sources, making systematic comparative studies a cumbersome task. Furthermore, comparative analyses benefit from close integration of derived data sets that cluster genes or organisms in a way that eases the expression of requests that clarify points of similarity or difference between species.

**Description:**

To support systematic comparative analyses of fungal genomes we have developed the e-Fungi database, which integrates a variety of data for more than 30 fungal genomes. Publicly available genome data, functional annotations, and pathway information has been integrated into a single data repository and complemented with results of comparative analyses, such as MCL and OrthoMCL cluster analysis, and predictions of signaling proteins and the sub-cellular localisation of proteins. To access the data, a library of analysis tasks is available through a web interface. The analysis tasks are motivated by recent comparative genomics studies, and aim to support the study of evolutionary biology as well as community efforts for improving the annotation of genomes. Web services for each query are also available, enabling the tasks to be incorporated into workflows.

**Conclusion:**

The e-Fungi database provides fungal biologists with a resource for comparative studies of a large range of fungal genomes. Its analysis library supports the comparative study of genome data, functional annotation, and results of large scale analyses over all the genomes stored in the database. The database is accessible at , as is the WSDL for the web services.

## Background

A large number of genome projects are under way, with about 670 genomes completely sequenced and more than 2,500 genomes still in progress (Genomes OnLine Database (GOLD) [1] statistic, accessed November 2007). Bacterial sequencing projects form the largest group of genome projects with about 1,800 completed or ongoing, followed by the eukaryotes with about 850 projects. Amongst the eukaryotes, about 200 fungal genomes are being sequenced, followed by protozoa and plants with about 140 and 130 sequencing projects, respectively. The large number of sequenced genomes can provide the basis for comparative genomics analyses, which have already proven invaluable for studying the evolution and genetic diversity of kingdoms, identifying species-specific genes and those conserved between genomes, or examining the expansion or contraction of protein families (e.g., [2-6]).

Not only are the fungi the most frequently sequenced kingdom within the eukaryotes, in addition the sequenced fungi have been selected to form clusters of related species, thus maximising their combined value for comparative genomics and evolutionary biology [7]. They also play an important role in medicine, agriculture and industry. This makes the fungi a prime candidate for a systematic comparative study of eukaryotic biology and evolution.

Comparative analyses can be used, amongst others, for the following analyses:

• Identification of species-specific proteins/protein families or those conserved in closely related species, which can help to analyse conservations in species exhibiting distinct phenotypes, e.g., growth habits, lifestyles, or pathogenicity;

• Study of genome redundancy in a range of related species, which can be used to analyse genome duplication;

• Study of contraction or expansion of gene/protein families;

• Identification of secreted proteins, which in pathogenic fungi could play important roles in host-pathogen interactions;

• Conservation of genes defined as essential for growth in *Saccharomyces cerevisiae *[8] in fungal genomes;

• Study of metabolic pathways in the fungi, analysis of conservation of components of pathways in fungal genomes; and

• Distribution, diversity and conservation of proteins with particular functional domains in related fungal genomes.

With the wealth of sequenced fungal genomes, the fungi can therefore not only serve as model organisms for eukaryotes [7], but could also provide an important setting for the development of techniques for comparative analysis of eukaryotes.

To facilitate comparative genomics, genomic data needs to be stored in multi-species databases instead of model genome databases capturing only data on a single genome [9,10]. For the fungi, a number of multi-genome data repositories are already available in which data generated by fungal genome sequencing projects is deposited. These data sources include SGD [11], the Fungal Genome Initiative (FGI) at the Broad Institute [12] or the Integrated Microbial Genome (IMG) resource provided at the JGI [13]. A large number of genomes are also available through Entrez [14]. Although these data sources store many fungi, the emphasis in their design is not primarily on a systematic comparison as such.

**Figure 1:**
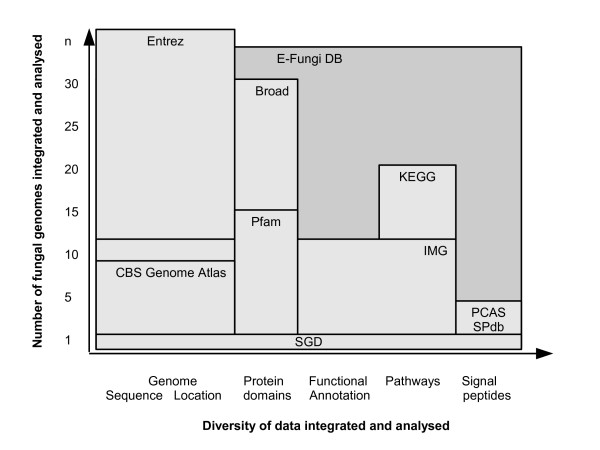
Overview of diversity of available databases.

Furthermore, a number of additional databases are available, specialising in particular kinds of data, some of which are placed in Figure 1 according to the diversity of data they integrate and the number of genomes they cover. These resources include the Gene Ontology project [15] providing functional annotation of proteins, the Pfam database [16] providing information on protein domains and families, KEGG [17], Reactome [18] and Metacyc [19] capturing information about pathways, as well as PCAS [20] and SPdb [21] storing predicted signal peptides. These specialised databases tend to contain only one particular kind of data but for a fairly large number of genomes. However, despite the large number of genomes integrated, as shown in Figure 1, most of these databases tend to cover only a limited number of fungal genomes. In addition to the types of data already mentioned, more and more functional genomics data sets are becoming available in various data sources.

Even though a number of multi-genome data sources are available, the distribution of the genomic data and additional data, such as functional annotation or pathway information, in heterogeneous data repositories, makes systematic comparisons of a large number of genomes a challenging task. To overcome the issues associated with the distribution of data in heterogeneous data repositories, and to facilitate comparative studies, a number of approaches have been taken to integrating a variety of different kinds of data for a large number of genomes. Databases that contain fungal among other genomes include Génolevures [22], IMG [13], Ensembl [23], the UCSC genome browser [24] and Entrez [14].

However, even though these are multispecies databases they do not provide analysis facilities powerful enough to carry out comparative analyses as mentioned above. This is due to the provision of predominantly gene-centred query facilities and visualisations that also tend to be species-centred in the sense that the analysis or search is focussed on a particular genome, and the results can then be related to other species, for example, by identification of orthologous proteins. This limitation makes the systematic comparative analysis of a large range of genomes a cumbersome task.

Here, we present e-Fungi, the first large-scale integrative repository of fungal genomes with an emphasis on supporting systematic comparative studies. To achieve this, e-Fungi integrates primary data obtained from a number of data sources and complements it with results of cluster analyses and other derived data that has been generated using large scale analyses of the genome data. The stored data can be analysed using a library of tasks that can be accessed using a web interface provided on the e-Fungi website [25] and as web services. With an emphasis on cluster-based analysis carried out over a range of genomes, e-Fungi represents a departure from gene-centric data sources and a move towards cluster-based data sources that provide better support for comparative studies.

## Construction and content

In this section the construction and content of the e-Fungi database are described. The data sources from which the primary data are obtained are introduced, as well as the processes that generate the derived data. Furthermore, an overview of the database schema is provided, the loading infrastructure introduced, and the library of analysis tasks presented.

### Data collection

#### Primary data

Four different types of primary data are obtained from a variety of repositories and integrated into the e-Fungi database: genomic data, Gene Ontology annotations, pathway data and EST data. Genomic data consists of the genome sequence with varying degrees of annotation. This annotation can include the prediction of genes with their introns, exons and predicted proteins, as well as their locations on contigs, supercontigs or chromosomes. Table 1 lists all the genomes integrated into the e-Fungi database with the data sources from which the data has been obtained. Other data has been obtained as follows:

**Table 1:** Genomes in e-Fungi with associated data sources

**Genome**	**Taxonomy**	**Pathogenicity**	**Growth form**	**Source**
*Phytophthora sojae*	Oomycete	plant pathogen	filamentous	JGI
*Phytophthora ramorum*	Oomycete	plant pathogen	filamentous	JGI
*Rhizopus oryzae*	Zygomycota – Mucorales	animal pathogen	filamentous	Broad
*Ustilago maydis*	Basidiomycete – Ustilaginomycota	plant pathogen	dimorphic	Broad
*Phanerochaete chrysosporium*	Basidiomycete – Homobasidiomycota	non pathogen	filamentous	JGI
*Schizosaccharomyces pombe*	Ascomycete – Schizosaccharomycetes	non pathogen	yeast – fission	Entrez
*Yarrowia lipolytica*	Ascomycete – Saccharomycetes	non pathogen	yeast – dimorphic	Entrez
*Saccharomyces paradoxus*	Ascomycete – Saccharomycetes	non pathogen	yeast	SGD
*Saccharomyces cerevisiae*	Ascomycete – Saccharomycetes	non pathogen	yeast	Entrez
*Saccharomyces mikatae*	Ascomycete – Saccharomycetes	non pathogen	yeast	SGD
*Saccharomyces kudriavzevii*	Ascomycete – Saccharomycetes	non pathogen	yeast	SGD
*Saccharomyces bayanus*	Ascomycete – Saccharomycetes	non pathogen	yeast	SGD
*Saccharomyces castellii*	Ascomycete – Saccharomycetes	non pathogen	yeast	SGD
*Candida glabrata*	Ascomycete – Saccharomycetes	animal pathogen	psuedo hyphae – dimorphic	Entrez
*Kluyveromyces waltii*	Ascomycete – Saccharomycetes	non pathogen	yeast	Entrez
*Saccharomyces kluyveri*	Ascomycete – Saccharomycetes	non pathogen	yeast	SGD
*Kluyveromyces lactis*	Ascomycete – Saccharomycetes	non pathogen	yeast	Entrez
*Eremothecium gossypii*	Ascomycete – Saccharomycetes	plant pathogen	filamentous	Entrez
*Candida albicans*	Ascomycete – Saccharomycetes	animal pathogen	psuedo hyphae – dimorphic	Entrez
*Debaryomyces hansenii*	Ascomycete – Saccharomycetes	non pathogen	yeast – dimorphic	Entrez
*Candida lusitaniae*	Ascomycete – Saccharomycetes	animal pathogen	yeast – dimorphic	Broad
*Coccidioides immitis*	Ascomycete – Eurotiomycetes	animal pathogen	filamentous	Broad
*Aspergillus oryzae*	Ascomycete – Eurotiomycetes	non pathogen	filamentous	Dogan
*Aspergillus niger*	Ascomycete – Eurotiomycetes	non pathogen	filamentous	JGI
*Aspergillus fumigatus*	Ascomycete – Eurotiomycetes	animal pathogen	filamentous	CADRE
*Aspergillus terreus*	Ascomycete – Eurotiomycetes	animal pathogen	filamentous	Broad
*Aspergillus nidulans*	Ascomycete – Eurotiomycetes	non pathogen	filamentous	Broad
*Stagonospora nodorum*	Ascomycete – Dothideomycetes	plant pathogen	filamentous	Broad
*Sclerotinia sclerotiorum*	Ascomycete – Leotiomycetes	plant pathogen	filamentous	Broad
*Botrytis cinerea*	Ascomycete – Leotiomycetes	plant pathogen	filamentous	Broad
*Trichoderma reesei*	Ascomycete – Sordariomycetes	non pathogen	filamentous	JGI
*Gibberella zeae*	Ascomycete – Sordariomycetes	plant pathogen	filamentous	Broad
*Magnaporthe grisea*	Ascomycete – Sordariomycetes	plant pathogen	filamentous	Broad
*Chaetomium globosum*	Ascomycete – Sordariomycetes	animal pathogen	filamentous	Broad
*Neurospora crassa*	Ascomycete – Sordariomycetes	non pathogen	filamentous	Broad
*Encephalitazoon cuniculi*	Microsporidia	animal pathogen	microsporidia	Entrez

• Gene Ontology annotation for *S. cerevisiae, S. pombe *and *C. albicans *has been obtained from SGD [11], Sanger GeneDB [26] and CGD [27].

• Pathway information including the assignment of pathways to proteins for *S. cerevisiae, S. paradoxus, S. mikatae, S. bayanus, E. gossypii, K. lactis, K. waltii, D. hansenii, C. albicans, C. glabrata, Y. lipolytica, S. pombe, N. crassa, M. grisea, A. nidulans, A. fumigatus, A. oryzae *and *E. cuniculi *has been obtained from KEGG [17].

• Expressed Sequence Tag (EST) data are obtained from the COGEME Phytopathogenic Fungi and Oomycete EST Database [28].

#### Derived data

**Figure 2:**
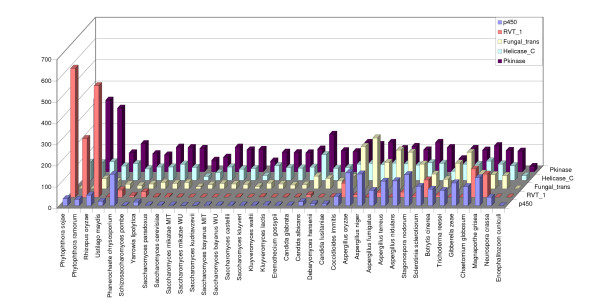
Distribution of 5 of the most frequently found Pfam domains.

**Figure 3:**
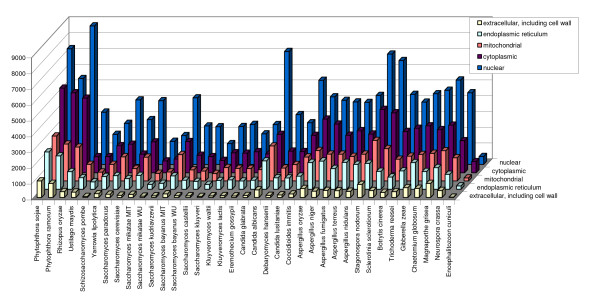
Distribution of the most frequently assigned PSort predictions.

**Figure 4:**
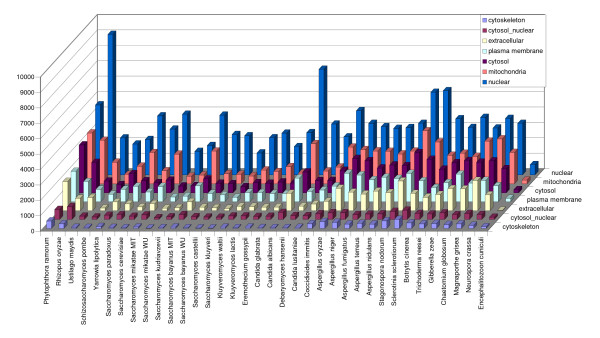
Distribution of the most frequently assigned Wolf-PSort predictions.

The following kinds of derived data are stored in the database:

• Clustering sequences from 36 fungal genomes: We compared 348,995 protein sequences from the 36 genomes integrated in e-Fungi (see Table 1) using BlastP [29] with an E-value cut-off of 10^-5^. This resulted in 47,342,483 hits. Markov Chain Clustering (MCL) [30] was then applied to generate clusters of similar proteins, using 2.5 as a moderate inflation value and 10^-10 ^as a comparatively strict E-value cut-off. This generated 23,724 clusters containing in total 282,061 sequences, while 66,934 sequences were singletons.

• Orthology assignments: To identify orthologous proteins between the 36 genomes, the BlastP results were analysed with OrthoMCL [31] using its default parameters (i.e., an E-value of 10^-5^). The analysis produced in total 30,084 clusters, with 5,406 of those containing just paralogues and 24,678 containing potential orthologous proteins. Out of these clusters of potential orthologues, 14,113 are unambiguous orthologue clusters, while 10,565 are ambiguous clusters with orthologues and recent paralogues.

• Domain assignments: To identify functional domains and other known sequence motifs, predicted proteins from all 36 genomes were scanned with the Pfam database release 18 [16] using hmmpfam [32]. A total of 6,260 different Pfam domains were identified in 196,425 proteins, using an E-value cut-off of 0.1. The distribution of 5 of the most frequently found Pfam domains among the genomes is shown in Figure 2.

• Protein localisation predictions: Protein sub-cellular localisations were predicted using SignalP [33], PSort [34] and Wolf-PSort [35] with the default parameters. Distributions of the most frequently assigned PSort and Wolf-PSort predictions among the genomes are shown in Figures 3 and 4.

All the generated data are integrated into the e-Fungi database using the loading infrastructure described below.

### Implementation

**Figure 5:**
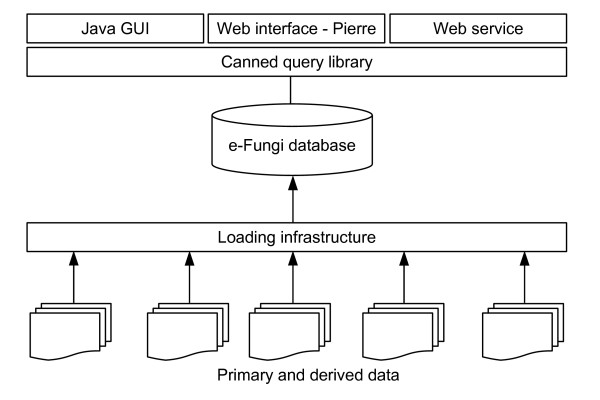
Overview of e-Fungi architecture.

The e-Fungi infrastructure consists of several components: the database itself, the population infrastructure, and the library of analysis tasks. An overview of the infrastructure is shown in Figure 5 and its components are introduced below.

#### Database schema

The Object Database Management System Versant FastObjects [36] is used to store the data integrated into e-Fungi. The database schema has been implemented using Java Data Objects (JDO), an industry standard interface-based abstraction of persistence. Using JDO for storing the data allows the direct implementation of the object data model without the need to map between the object model and, for example, a relational database model. Such a mapping often results in a less intuitive representation of the data.

Using an object data model in combination with an object-oriented programming language, such as Java, also enables a tighter integration of analysis tasks with the stored data. Complex queries that analyse a large variety of different types of data can, therefore, be realised in a fairly intuitive manner.

The database schema can be divided into different parts, modelling the different types of data introduced above. The parts of the schema for genomic sequences, annotations, pathways and ESTs are based on published models [17,28,37,38]. The part of the schema modelling the derived data is introduced in more detail in the following.

Results of the MCL and OrthoMCL cluster analyses consist of an identifier for each cluster and the assignments of proteins to clusters, captured in the classes MclCluster and OrthoMclCluster. To be able to retrieve the MCL cluster or the OrthoMCL cluster for a particular protein, the class Protein has an association with both MclCluster and OrthoMclCluster.

The results of the predictions of protein sub-cellular localisations are captured following a similar approach for all three different prediction methods. Each prediction method can have a number of different outcomes, e.g., golgi, cytoplasmic, or plasma membrane. These are captured in PSortPrediction, WolfPSortPrediction and SignalPPrediction. Each prediction has a 0-to-many association with Protein, enabling the retrieval of all proteins with a particular predicted localisation. However, not only are the final predictions provided as a result of the analyses, so are a number of scores associated with the predictions. Scores returned by each prediction analysis are captured in PSortResult, WolfPSortResult and SignalPResult, which have a 1-to-1 association with the protein for which the prediction has been made. The scores are captured as provenance information associated with each analysis, thereby recording all the information contained in the report provided as a result of each analysis.

#### Loading infrastructure

**Figure 6:**
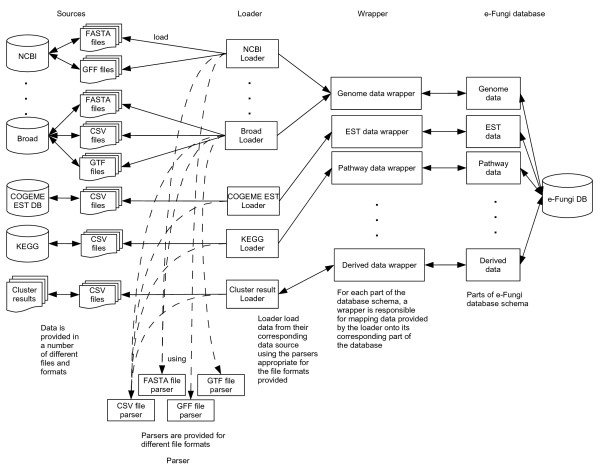
**Loading infrastructure**. Schematic overview of the loading infrastructure employed to integrate primary and derived data into the e-Fungi database.

A loading infrastructure has been developed to integrate data from a variety of data sources, as listed in Table 1, and map the information onto the e-Fungi database schema. The infrastructure consists of 3 general modules, the loaders, parsers and wrappers (see Figure 6). The loaders are specific to each data source and data format. For example, the genomic data loader for data from the Broad Institute gathers the contigs, genes and protein sequence data from 3 separate FASTA files and relates the data to information provided in other files of different formats. Data from each source is parsed into a generalised format that can be processed by the loader using the respective parsers for each of the available data formats (e.g., FASTA, GTF, GFF). The wrappers are responsible for creating and linking objects, e.g., when loading data on a protein the wrapper will create a Protein, create a PrimaryPolypeptide sequence, and link these together.

The loading infrastructure is designed to minimise maintenance and ensure extensibility of the database. Each module protects the others from sections that are prone to changes. For example, changes in the source, e.g., changes in location, format or method of access, will only require modifications of the loaders without affecting the existing parsers or wrappers. The parsers, on the other hand, act as tools for the loaders and can be easily improved or added as required. For the database, wrappers provide a layer of protection that allows the schema to be changed without the need to modify existing loaders or parsers. This allows the database to be extended easily to include further kinds of data without the need to rebuild the loading infrastructure.

#### Library of analysis tasks

The data stored in the e-Fungi database can be analysed using pre-determined analysis tasks that can be parameterised, so-called canned queries. More than 90 queries are currently available, varying in their complexity from simple retrieval tasks to complex analysis tasks. Similar queries based on the type of data analysed are grouped together (see Table 2 for an overview of the categories). Providing pre-determined analysis tasks as a means to explore and analyse the stored data might seem quite limiting at first. However, as shown in the next section, it allows complex analysis tasks to be provided that are beyond simple keyword- or identifier-based retrieval of stored data.

**Table 2:** Canned query groups currently provided

**Canned query group**	**Canned query group**
Annotation of proteins in clusters	Queries in this group retrieve annotation of all the proteins in particular clusters. The annotation consists of PSort, Wolf-PSort and SignalP predictions, as well as GO annotations, Pfam domains, Enzyme annotation and pathways for each protein, as well as its assignment to a particular MCL and OrthoMCL cluster. The clusters can either be chosen by providing an identifier of a particular cluster or they can be based on the proteins they contain, such as proteins with a particular GO annotation or a particular cellular localisation as predicted by PSort or Wolf- PSort.
Cellular localisation analysis	This group of queries retrieves the cellular localisation for proteins as predicted by PSort and Wolf-PSort. It also retrieves proteins with a particular predicted cellular localisation.
EST analysis	Collection of general EST analyses. Information available include group/hierarchy structure of ESTs and genes as well as number of homologs of genes in all genomes in the database.
Essential yeast genes cluster analysis	Queries to retrieve Mcl Clusters containing proteins of a given genome and proteins of essential or non-essential yeast genes.
Essential yeast genes orthology analysis	This group of queries analyses clusters containing a given genome and proteins of essential or non-essential yeast genes in terms of the number of genomes present in those clusters.
Functional annotation analysis	Queries in this group enable the retrieval of Gene Ontology or Pfam annotation for a given protein, or the retrieval of proteins with a given annotation.
Genomics analysis	Collection of queries for general genomic analyses, such as retrieving the exons of a particular gene.
MCL cluster analysis	Queries in this group provide a general analysis of the MCL clusters in the database. Clusters containing proteins of a given genome, or a group of genomes, such as plant pathogens or filamentous fungi, can be retrieved. Furthermore, clusters that contain more or less than a given percentage of proteins of a given genome can also be obtained.
OrthoMCL cluster analysis	This group of queries provide a general analysis of the OrthoMCL clusters in the database. The queries in this group are similar in scope to the queries in the MCL cluster analysis group.
Pathway analysis	Queries provided in this group retrieve pathways and enzyme annotations for a particular protein as well as all the proteins in a given pathway or with a particular enzyme annotation.
Redundancy analysis	The query in this group analyses the redundancy in a given species. Genome redundancy is determined by counting the number of proteins of that given genome in MCL clusters.
Secretome analysis	To retrieve the SignalP prediction for a given protein or proteins with a given SignalP prediction, i.e., secretory or non-secretory proteins, queries in this group can be used.
Transcript abundance	Collection of queries for transcript abundance analyses. These queries enable the identification of genes that may be highly expressed under a particular growth condition. Information of these genes and conditions can also be retrieved.

## Utility

The data stored in e-Fungi can be accessed through a web interface and web services, which have been generated using Pierre [39]. A Java Graphical User Interface (GUI), described elsewhere [38], is currently only used locally, but can be made available on request.

### Web interface access

The database can be accessed through the e-Fungi web site [25] by either following the link 'Connect to the database' or by choosing the category 'Database' in the menu on the left hand side of the page and choosing the 'Connect to the database' link. In both cases, a further link to the WSDL describing the web services is also provided. The web services are introduced later.

#### Browse

Browse provides an overview of the genomes stored in the database, but can also be used as an entry point to explore and analyse the data by following either of the two different kinds of links provided: (i) navigational links, and (ii) links to analysis tasks. The former, for example, enable the retrieval of contigs or chromosomes for a particular genome, whereas the latter link to analysis tasks provided in the canned query library using the chosen entry, e.g., a particular genome, as input.

#### Simple Search

**Figure 7:**
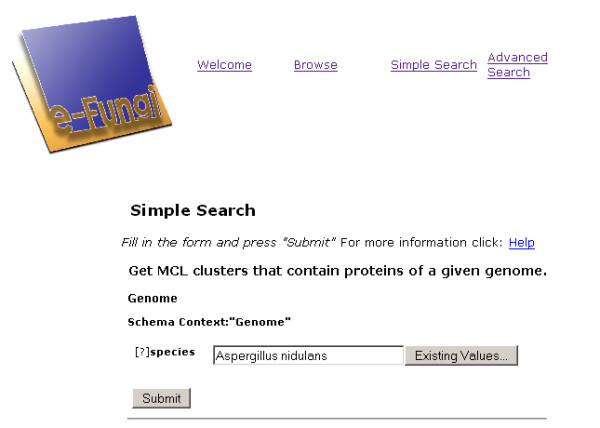
**Screenshot of parameterisation of a canned query**. Screenshot of the web interface showing the parameterisation of the query 'Get clusters with proteins of a given genome.', which is part of the group 'MCL cluster analysis'.

**Figure 8:**
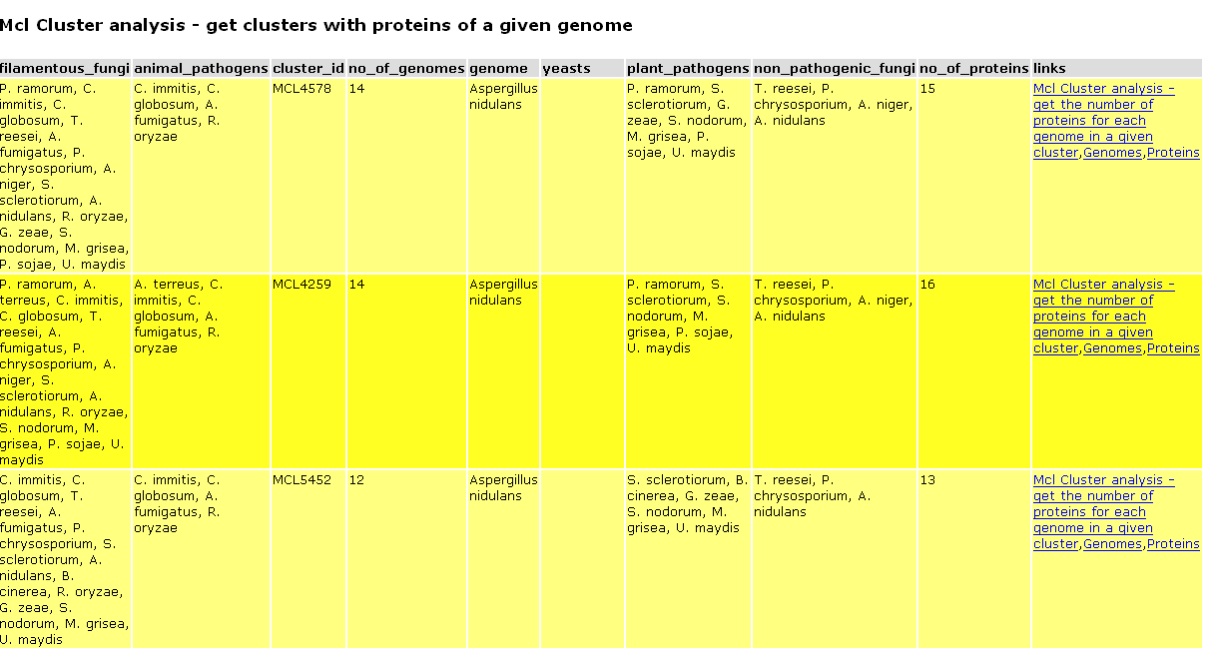
**Screenshot of the query result**. Screenshot of the web interface showing a subset of the MCL clusters with *Aspergillus nidulans *proteins. The clusters shown are the three clusters containing only proteins of filamentous genomes and no yeast like genomes, whereas all the remaining 7593 contain both.

The Simple Search feature exposes the tasks provided in the canned query library mentioned above. Documentation for each query can be found under the category 'Documentation' in the menu on the left hand side of the e-Fungi web site or by following the 'Help' link provided on each query form (see Figure 7). Information includes the type of input required, a number of example inputs, and a description of the output provided by the query. Furthermore, information on the runtime of long running queries is also provided. The canned queries provided to support the comparative analyses listed in the background section are introduced in the following to illustrate the utility of the e-Fungi database:

1. *Identification of species-specific protein families or those conserved in closely related species*. The queries 'Get MCL clusters with proteins of a given genome' or 'Get OrthoMCL clusters with proteins of a given genome', which can be found in the group 'MCL cluster analysis' and 'OrthoMCL cluster analysis', respectively, can be used to retrieve all clusters containing proteins of a particular genome and perhaps identify clusters containing only paralogues of the chosen genome, i.e., possible species-specific proteins. To run a query, the appropriate query category is chosen, e.g., MCL clusters or OrthoMCL clusters. From the list of canned queries in the chosen group, the canned query of interest is selected and the user is presented with a form for the required input parameters (e.g., Figure 7). For some of the input parameters, an existing value feature exists, enabling users to choose a value from a list of possible values. For other parameters, the Advanced Search feature can be used to retrieve the exact value, such as for a particular Gene Ontology Annotation or Pfam domain, as illustrated later. With the input parameters provided, the query can be executed and the results displayed (see Figure 8). Some of the result reports provide navigation in the form of links, similar to the navigation in Browse, for further exploration and analysis of the results.

2. *Contraction or expansion of protein families*. The query 'Get all MCL clusters with more than a given percentage of proteins of a given genome' can be used to identify outlying clusters. The query is part of the group 'MCL cluster analysis' and has a counterpart in the group 'OrthoMCL cluster analysis'. To identify protein families that are conserved in genomes exhibiting a certain phenotype, the query 'Get MCL clusters containing proteins of a group of genomes' or its counterpart that analyses OrthoMCL clusters can be used. A group of genomes can be specified by their exhibited phenotypes, such as growth form or pathogenicity. Analyses to identify species-specific protein families or those that are conserved in related species with a particular phenotype, as well as studies of contraction or expansion of protein families, have been part of recent comparative studies [3,40-42].

3. *Genome redundancy in a range of related species, illustrating the importance of genome duplication *[43,44]. The canned query 'Get the number of paralogues for all clusters containing proteins of a given genome' that can be found in the group 'Redundancy analysis' can be used.

4. *Identification of secreted proteins, which in pathogens could play important roles in host-pathogen interactions*. This analysis can be aided by executing either of the following canned queries 'Get secretory proteins for a given genome', which is part of the group 'Secretome analysis', or 'Get annotation for proteins of a given genome in MCL/OrthoMCL clusters with secretory proteins'. The queries retrieving the annotation of proteins in MCL or OrthoMCL clusters are part of the group 'Annotation of proteins in clusters'.

5. *Conservation of genes defined as essential for growth in Saccharomyces cerevisiae *[8]* among fungal genomes*. This analysis is supported by a number of queries that can be found in the groups 'Essential yeast genes cluster analysis' and 'Essential yeast genes orthology analysis'. Similar studies using the essential genes identified in *Candida albicans *have been reported in [7].

6. *Conservation of components of metabolic pathways among fungal genomes *[45]. Again, this analysis is supported by a number of canned queries, such as 'Get proteins that are in the same (KEGG reference) pathway as a given protein' of the group 'Pathway analysis', which retrieves all the proteins that are known to participate in a particular pathway. To analyse newly sequenced genomes and identify proteins that could potentially be part of a pathway, the query 'Get annotation for proteins of a given genome in the same MCL/OrthoMCL clusters as proteins in a given pathway', part of the 'Annotation of proteins in clusters' group, can be used.

#### Advanced Search

**Figure 9:**
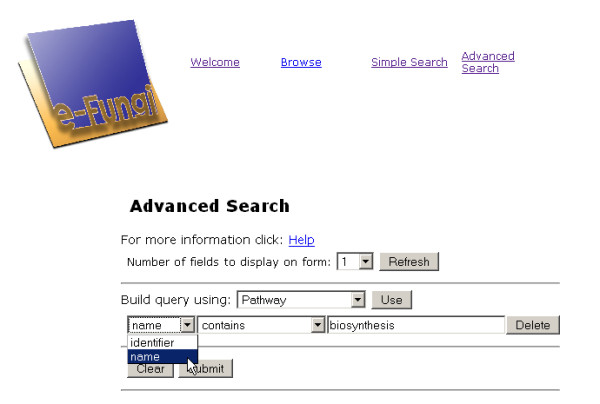
**Screenshot of Advanced search**. Screenshot of the Advanced search feature of the web interface. This feature enables the filtering of objects of a particular type and can be used to retrieve the exact value of names or identifiers of which only the beginning or end is known.

The Advanced Search feature can be used to retrieve entries for which a property value or a range of property values can be specified. The user specifies the type of entry to be retrieved and the filters that the returned entries have to match. Similar to the Simple Search, a form is provided requesting input parameters for the Advanced Search. The example of an Advanced Search shown in Figure 9 retrieves all the biosynthesis pathways, i.e., all the KEGG pathways the name of which ends in 'biosynthesis'.

### Web service access

Programmatic access to all the simple and advanced search facilities is provided by a web service interface. This enables the integration of e-Fungi web services with other web services to build complex workflows for data analysis and visualisation.

**Figure 10:**
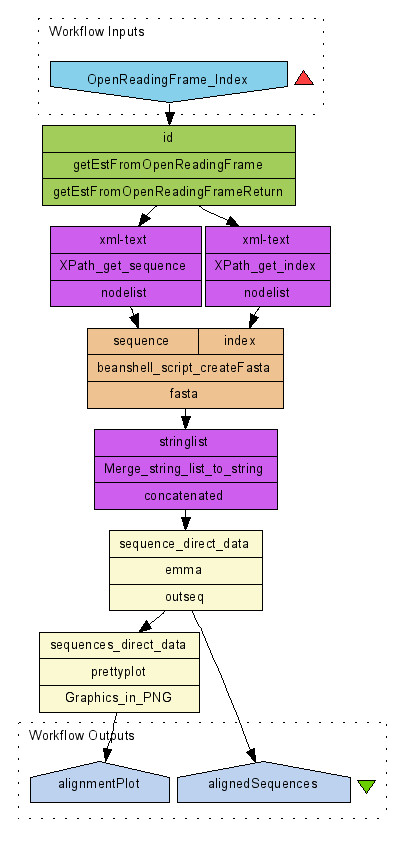
**Sample workflow**. Workflow schema describing multi-sequence alignment and visualisation using web services.

A simple workflow example, implemented in Taverna [46], is shown in Figure 10. In this example, the ESTs representing an Open Reading Frame (ORF) are aligned. The workflow, built using web services from e-Fungi and EBI SOAPLab [47], retrieves the ESTs (that represent the ORF of interest), and generates a set of aligned EST sequences and an alignment plot. Firstly, the e-Fungi web service operation, 'getEstFromOpenReadingFrame' is used to retrieve all the ESTs that represent the ORF of interest. The results are then parsed, the required information extracted (using tools in Taverna) and passed to the web service operation 'emma' (from EBI SOAPLab) that performs multiple sequence alignments. The results are then sent to the operation 'prettyplot' (from EBI SOAPLab) to generate an alignment plot, highlighting the aligned sections for the group of ESTs. The WSDL for the e-Fungi web service used within this workflow is:

<wsdl:definitions targetNamespace="urn:uk.org.efungi"

   xmlns:soapenc="http://schemas.xmlsoap.org/soap/encoding/">

   <wsdl:message name="getEstFromOpenReadingFrameRequest">

      <wsdl:part name="id" type="soapenc:string"/>

   </wsdl:message>

   <wsdl:message name="getEstFromOpenReadingFrameResponse">

      <wsdl:part name="getEstFromOpenReadingFrameReturn" type="soapenc:string"/>

   </wsdl:message>

   <wsdl:portType>

      <wsdl:operation name="getEstFromOpenReadingFrame" parameterOrder="id">

         <wsdl:input message="impl:getEstFromOpenReadingFrameRequest"

            name="getEstFromOpenReadingFrameRequest"/>

         <wsdl:output message="impl:getEstFromOpenReadingFrameResponse"

            name="getEstFromOpenReadingFrameResponse"/>

      </wsdl:operation>

   </wsdl:portType>

</wsdl:definitions>

Two different kinds of web services are provided for the simple searches: (i) specific and (ii) generic. The specific web service offers users a separate operation for each individual canned query, while the generic service provides users an all-in-one operation that is able to access all the available canned queries. All results returned from the web services are formatted in XML to ease parsing. Supporting operations are also provided to aid the usage of the web service. For example, the web service operation 'identifyAdvancedSearchCollections' returns all the available types of data that support the Advanced Search. The e-Fungi web service is deployed using Axis [48] and can be accessed via Taverna (among other methods) by using Taverna's 'WSDL scavenger' feature.

### Case study – Using e-Fungi to investigate fungal cytochrome P450 proteins

The case study presented in this section investigates the distribution, diversity and conservation of proteins with particular functional domains among related fungal genomes [3,41-43,49].

Cytochrome P450 proteins form a superfamily of proteins which are found in many organisms, including bacteria, fungi, plants and mammals. They are monooxygenase enzymes that catalyse bioconversion processes. These include the degradation of complex biopolymers, such as the breakdown of lignin by *Phanerochaete chrysosporium *[50], and the production of secondary metabolites. In order to compare the P450omes [50] of different fungal species, we used the query 'Get proteins with a given Pfam annotation', which can be found in the group 'Functional annotation analysis', entering the accession number PF00067. The result of this query is a list of proteins in which this motif has been identified, along with the E-Value and score associated with each identification as well as the MCL and OrthoMCL cluster in which the protein has been placed. The numbers of P450 proteins identified in each fungal species (see Table 3) appear to be in agreement with previously published results for *A. oryzae *(149 P450 proteins), *A. nidulans *(102), *A. fumigatus *(72) [51] and *P. chrysosporium *(150) [50].

**Table 3:** Clustering of 450 proteins

**Species**	**# P450 proteins**	**# OrthoMCL clusters**	**# Proteins not in OrthoMCL clusters**
*Phytophthora sojae*	35	18	6
*Phytophthora ramorum*	31	17	3
*Rhizopus oryzae*	49	8	4
*Ustilago maydis*	20	15	0
*Phanerochaete chrysosporium*	150	25	10
*Schizosaccharomyces pombe*	2	2	0
*Yarrowia lipolytica*	17	7	1
*Saccharomyces paradoxus*	3	3	0
*Saccharomyces cerevisiae*	3	3	0
*Saccharomyces mikatae*	3	3	0
*Saccharomyces kudriavzevii*	2	2	0
*Saccharomyces bayanus*	3	3	0
*Saccharomyces castellii*	3	3	0
*Saccharomyces kluyveri*	3	3	0
*Kluyveromyces waltii*	3	3	0
*Kluyveromyces lactis*	5	5	0
*Eremothecium gossypii*	3	3	0
*Candida glabrata*	3	3	0
*Candida albicans*	19	7	0
*Debaryomyces hansenii*	9	6	0
*Candida lusitaniae*	8	7	0
*Coccidioides immitis*	44	32	6
*Aspergillus oryzae*	155	86	28
*Aspergillus niger*	150	86	20
*Aspergillus fumigatus*	72	50	5
*Aspergillus terreus*	116	67	25
*Aspergillus nidulans*	119	79	15
*Stagonospora nodorum*	148	83	37
*Sclerotinia sclerotiorum*	92	70	13
*Botrytis cinerea*	79	53	18
*Trichoderma reesei*	71	43	10
*Gibberella zeae*	107	65	14
*Chaetomium globosum*	89	68	14
*Magnaporthe grisea*	133	67	30
*Neurospora crassa*	39	33	2
*Encephalitazoon cuniculi*	0	0	0

The distribution of P450 proteins amongst the fungi is clearly unequal. The Hemiascomycetes and the Schizosaccharomycete *Sz. pombe *have far fewer P450 proteins than filamentous Ascomycetes and the Basidiomycetes. However, there are also large differences between different filamentous Ascomycetes and Basidiomyctes. Analysis of the result with respect to the placement of P450 proteins in OrthoMCL clusters reveals differences between several Pezizomycotina and *P. chrysosporium*. Firstly, for the Pezizomycotina species, there are more P450 proteins that are not part of an OrthoMCL cluster than there are for *P. chrysosporium*. The second difference is that *P. chrysosporium *P450 proteins are found in far fewer OrthoMCL clusters than the P450 proteins from the Pezizomycotina species. This difference is due in part to a few highly duplicated *P. chrysosporium *genes. Using the query 'Get annotation for proteins in a given OrthoMCL cluster' from the group 'Annotation of proteins in clusters', for example, shows that OrthoMCL cluster ORTHOMCL3134 contains 32 P450 *P. chrysosporium *paralogues and no proteins from any other species, while cluster ORTHOMCL190 contains 53 proteins including 14 *P. chrysosporium *proteins. In summary, our analysis identifies enormous differences in the P450omes of different fungal species. It is clear that budding yeasts and fission yeasts possess much smaller P450omes than Pezizomycetes, Basidiomycetes and Zygomycetes. More detailed analysis of five fungal species with large P450omes demonstrates that those of the four Pezizomycotina species appear to possess greater sequence diversity and less tandem duplication.

## Discussion

Queries provided by e-Fungi are focussed on the analysis of biological or evolutionary phenomena, such as gene duplication, or expansion and contraction of protein families in related species, rather than on sequence level comparisons of genomes, genes and proteins. Even though the clusters, forming the foundation for the majority of analyses provided, are created based on sequence similarity of proteins, the queries are not limited to retrieval of the results of those sequence-based analyses, but instead correlate cluster information with additional information, such as functional annotation, or prediction of protein sub-cellular localisations or identified Pfam motifs.

A number of data repositories support comparative genomics analysis, for example, the UCSC Genome Browser [24,52], NCBI [14], and Ensembl [23,53,54], all of which integrate a wide variety of genomes from different kingdoms, coliBase/xBase [55,56], Microbase [57], MolliGen [58] and the Comprehensive Microbial Resource (CMR) [59], which are data sources dedicated to bacterial comparative genomics. Furthermore, there is the Integrated Microbial Genomes (IMG) system [13], integrating a large number of microbial genomes, amongst them a smaller number of eukaryotes, and Génolevures [22] which contains 14 hemiascomycetous yeasts. These data repositories differ with respect to the number and diversity of genomes they cover, but also in the kinds of data integrated in addition to sequence data, and the analyses they provide.

Ensembl, NCBI, coliBase/xBase, CMR and Microbase integrate predominantly sequence data and provide comparative analyses based on nucleotide sequence similarities and orthologous proteins. In addition to sequence data, Génolevures integrates pathway and Pfam data, CMR also captures functional annotation and pathways, MolliGen provides pathway data, and both UCSC and IMG integrate amongst other data Pfam, functional annotation and pathway data, all of which have also been integrated into e-Fungi. However, despite the differences in the number of genomes and types of additional data integrated, search facilities tend to be quite similar, and limited to sequence similarity- or keyword-, identifier- or name-based searches for retrieval of the stored data. Such search facilities tend to be straightforward and self-explanatory to use, but less suitable for complex analyses of stored data, than those provided by e-Fungi.

However, in addition to data retrieval and sequence based comparisons, NCBI, MolliGen, Génolevures and IMG provide analyses that are aimed at understanding molecular evolution and are to some extent similar in scope to analyses provided by e-Fungi. Such analyses include the study of conservation of proteins between genomes or groups of genomes, as well as the conservation of pathways. However, these analyses are provided by bespoke analysis tools that are not part of the general query and analysis infrastructure, unlike in e-Fungi. Such tools include TaxPlots provided by the NCBI [14], Phylogenetic Profiler and Abundance Profiler provided by IMG [13], or the multi-proteome differential analysis facility provided by MolliGen [58]. Using bespoke tools for complex analyses is limiting in terms of scalability, as new tools have to be developed to provide complex analyses of different kinds. As complex analyses are part of the e-Fungi query and analysis infrastructure, new queries analysing different kinds of data can easily be added. The e-Fungi database integrates and makes available various of the data sets that have been used in previous comparative studies (e.g. [40,42,43]) but that have not typically been central to genomic databases. With its cluster-based genome comparison analyses, its integration of a variety of other kinds of information in addition to sequence and orthologue data, and its complex analysis tasks, e-Fungi moves away from sequence-based comparative genomics data sources that can predominantly be accessed by keyword or gene identifier-based queries.

The e-Fungi database is updated and extended in the form of themed releases, with 'Sequence' and 'Functional annotation' being the first two releases, and 'Functional genomics' the next release scheduled for the end of 2007. Not only are new types of data and new queries added according to the theme of the release, but also new genomes are added. For each release, all the derived data, including clustering and PFAM analysis, is regenerated and updated.

## Conclusion

The e-Fungi database integrates a large number of diverse fungal genomes and complements the wealth of genomic data with derived data generated by a range of analyses performed on the genomic data of all the genomes. The e-Fungi database is unique in the diversity of data that it provides for the large number of genomes it integrates. It is also unique in terms of the extensive canned query library for the analysis of the stored data it provides. The canned queries are motivated by recent comparative studies carried out to improve our understanding of evolutionary biology.

## Availability and requirements

The e-Fungi database can be accessed freely at . e-Fungi WSDL files can also be obtained from the website.

## Authors' contributions

CH and HMW implemented the e-Fungi infrastructure. CH, HMW, IA, MC wrote the initial draft. NWP and MR provided feedback on the initial draft. CH has revised the draft. DMS, MR, SJH, NJT, SGO and NWP provided input on the development and direction of the warehouse. All authors have read and approved the final manuscript.
